# A review of biomarkers in the context of type 1 diabetes: Biological sensing for enhanced glucose control

**DOI:** 10.1002/btm2.10201

**Published:** 2020-12-09

**Authors:** Kelilah L. Wolkowicz, Eleonora M. Aiello, Eva Vargas, Hazhir Teymourian, Farshad Tehrani, Joseph Wang, Jordan E. Pinsker, Francis J. Doyle, Mary‐Elizabeth Patti, Lori M. Laffel, Eyal Dassau

**Affiliations:** ^1^ Harvard John A. Paulson School of Engineering and Applied Sciences, Harvard University Cambridge Massachusetts USA; ^2^ Sansum Diabetes Research Institute Santa Barbara California USA; ^3^ Department of Nanoengineering University of California San Diego La Jolla California USA; ^4^ Joslin Diabetes Center, Harvard Medical School Boston Massachusetts USA

**Keywords:** automated insulin delivery, biosensors, measurement, medical devices, nanobiology, type 1 diabetes

## Abstract

As wearable healthcare monitoring systems advance, there is immense potential for biological sensing to enhance the management of type 1 diabetes (T1D). The aim of this work is to describe the ongoing development of biomarker analytes in the context of T1D. Technological advances in transdermal biosensing offer remarkable opportunities to move from research laboratories to clinical point‐of‐care applications. In this review, a range of analytes, including glucose, insulin, glucagon, cortisol, lactate, epinephrine, and alcohol, as well as ketones such as beta‐hydroxybutyrate, will be evaluated to determine the current status and research direction of those analytes specifically relevant to T1D management, using both in‐vitro and on‐body detection. Understanding state‐of‐the‐art developments in biosensing technologies will aid in bridging the gap from bench‐to‐clinic T1D analyte measurement advancement.

## 1 INTRODUCTION

Type 1 diabetes (T1D) is an autoimmune disease that results in loss of insulin‐producing beta cells of the pancreatic islets. The resulting absolute insulin deficiency causes hyperglycemia (high glucose levels), as well as changes in protein and lipid metabolism.^1^ As a result, individuals with T1D require uninterrupted exogenous insulin therapy to maintain their health and prevent severe metabolic complications, such as ketoacidosis. Currently, there are over 34.2 million people globally living with T1D, including over 500,000 people who are less than 15 years old.^1^, ^2^ Each year, 11.7 million people are diagnosed with T1D globally, while nearly 90,000 children are diagnosed worldwide.^1^, ^2^, ^3^ Less than one‐third of people living with T1D are consistently achieving target blood glucose levels in the range of 70–180 mg/dl.^4^ Glycemic targets generally relate to a hemoglobin A1c goal of less than 7%, or less than 58 mmol/mol for youth and less than 53 mmol/mol for adults; from 2016 to 2018, these goals were achieved by only 17% and 21% for youth and adults, respectively.^4^, ^5^ In the United States, T1D accounts for over 16 billion in healthcare expenditures and lost income annually.^6^

Individuals without T1D are able to maintain glucose levels within a normal (safe) range, between 70 and 140 mg/dl. This tight range is possible because the pancreatic beta cells within the islets of Langerhans secrete insulin at a constant low level (basal insulin secretion) and at higher levels intermittently in response to elevations in blood glucose, such as after meals, and reduce secretion when glucose levels fall. Moreover, alpha cells in the pancreas secrete glucagon when blood glucose levels are falling, as can occur with fasting and/or exercise, in order to prevent hypoglycemia (low glucose levels). Individuals with T1D have an absolute insulin deficiency. They depend on insulin either via injection or infusion via pump for control of glucose to prevent serious acute complications, such as ketoacidosis, as well as chronic complications, such as vision loss. However, the precise dosing of insulin required to match body metabolism is highly challenging, often resulting in either hyperglycemia or hypoglycemia. In addition, glucagon response to insulin‐induced hypoglycemia is impaired soon after the development of T1D, presenting further challenges to maintain tight glucose control.^7^ Maintenance of glucose control is important to reduce the frequency of long‐term microvascular complications, including retinopathy, neuropathy, and nephropathy, as well as macrovascular cardiovascular complications. These macrovascular cardiovascular complications include premature atherosclerosis, the main driver of excess mortality, causing coronary artery disease and myocardial infarction, cerebrovascular disease and stroke, and peripheral vascular disease.^8^ Beyond glucose, insulin deficiency impacts other aspects of systemic metabolism, such as increasing production of ketones and additional alterations in lipid and protein metabolism.^8^, ^9^

While technology such as continuous glucose monitoring (CGM) and advanced automated insulin delivery (AID) systems have been very helpful in improving control of glucose and prevention of hypoglycemia, the burden of self‐management remains very high for patients, their families, and multidisciplinary care teams.^10^ Individuals with T1D must understand many aspects of physiology related to glucose metabolism in order to choose an insulin dose which that their physiologic needs several times per day. This process is challenging, and requires an individualized approach. For example, even with the most modern and AID systems, insulin dosing before meals needs to take into account meal size and macronutrient composition, as well as current glucose level, in order to obtain optimal results; increased carbohydrate content requires more insulin, but this may be modified by fat and fiber content and other factors that influence absorption of the meal. Further adjustments in real‐time, such as reducing or increasing insulin doses still require user intervention. Physical activity can reduce blood glucose levels, requiring insulin dose reduction. Likewise, illness and stress can increase insulin requirements, but these are difficult to quantify and anticipate, making insulin adjustments challenging.

These clinical needs have motivated clinicians, scientists, and engineers to collaborate and develop technologies that can assist individuals with T1D to maintain safe glucose levels. In the past decade, major progress has been achieved in both glucose monitoring and insulin delivery. Wearable or implanted glucose monitors provide nearly instantaneous feedback (e.g., every 5 min) of interstitial fluid (ISF) glucose levels using a subcutaneous electrode and, with some devices, alerts for high or low glucose values.^11^ Intensive insulin therapy using either multiple daily injections or continuous subcutaneous insulin infusion pumps, have allowed more physiologic delivery of insulin.^12^ In general, these approaches rely on two major components of insulin therapy: (1) background or “basal” insulin secretion—the amount needed to maintain metabolism even if not eating and (2) meal‐related insulin secretion (termed “bolus” insulin). Moreover, the development of long‐acting insulins, which can mimic basal (background) insulin secretion, and rapid‐acting or ultra‐short‐acting insulins, which can be used for meal insulin delivery to more closely match the timing of food absorption, have been critical to allow more precise insulin administration and increase flexibility of meal timing and content.^13^ Development of smaller or implantable CGM, rapid‐acting insulins, smaller pumps, dual hormone pumps, and mobile apps have enabled the development of AID or artificial pancreas (AP) systems, and are likely to greatly improve care and quality of life for those with T1D.^14^ Further research of strategies to deliver insulin directly into the portal circulation and to engineer beta cells for transplantation may yield transformative therapies in the future.

Unfortunately, using CGM‐delivered glucose signals alone in a pump does not permit rapid or adequate response to glycemic variability due to unanticipated events, such as unannounced meals or exercise, stress, and illness, or over or under‐calculation of insulin dosing. Furthermore, the high intra‐ and intersubject variability in insulin pharmacokinetic dynamics affects the estimation of insulin time‐to‐peak and metabolic clearance rates.^15^ Beyond insulin, other hormones and metabolites can influence insulin responsiveness (e.g., cortisol) or serve as an indicator of physiological changes (e.g., increased lactate with exercise, increased ketones with interruption of insulin delivery or illness, etc.). Therefore, the inclusion of additional hormones as inputs for T1D control may help to balance the effects of delivered insulin with those of other hormones that contribute to glucose metabolism. Strong efforts have been directed over the past five decades toward developing advanced sensing systems for monitoring diabetes biomarkers.^16^, ^17^, ^18^, ^19^ This activity has led to a paradigm shift from blood glucose meters to CGM devices, and to the next‐generation of wearable minimally‐ and noninvasive platforms. By leveraging the remarkable advances in wearable sensor technology and introducing additional analyte signals obtained via minimally and/or noninvasive biosensors, potentially in combination, the physiological function of a healthy metabolic state without diabetes may be more effectively bio‐mimicked, ultimately improving glycemic control.

This review describes the ongoing investigation of developing biomarker analyte measures that can be used in T1D management. Recent technological advances in transdermal biosensing offer remarkable opportunities to move from research laboratories to clinical point‐of‐care (POC) applications. A range of analytes, including glucose, insulin, cortisol, lactate, beta‐hydroxybutyrate (BOHB), epinephrine, and alcohol will be evaluated in the context of T1D control. In an effort to determine the current status and research direction of feasible T1D analytes, on‐body and in‐vitro detection techniques are reviewed. It is believed that a thorough analysis of state‐of‐the‐art developments in biosensing technologies will aid in bridging the gap from bench‐to‐clinic T1D analyte advancement.

## 2 BIOMARKERS AND ANALYTES

**FIGURE 1:**
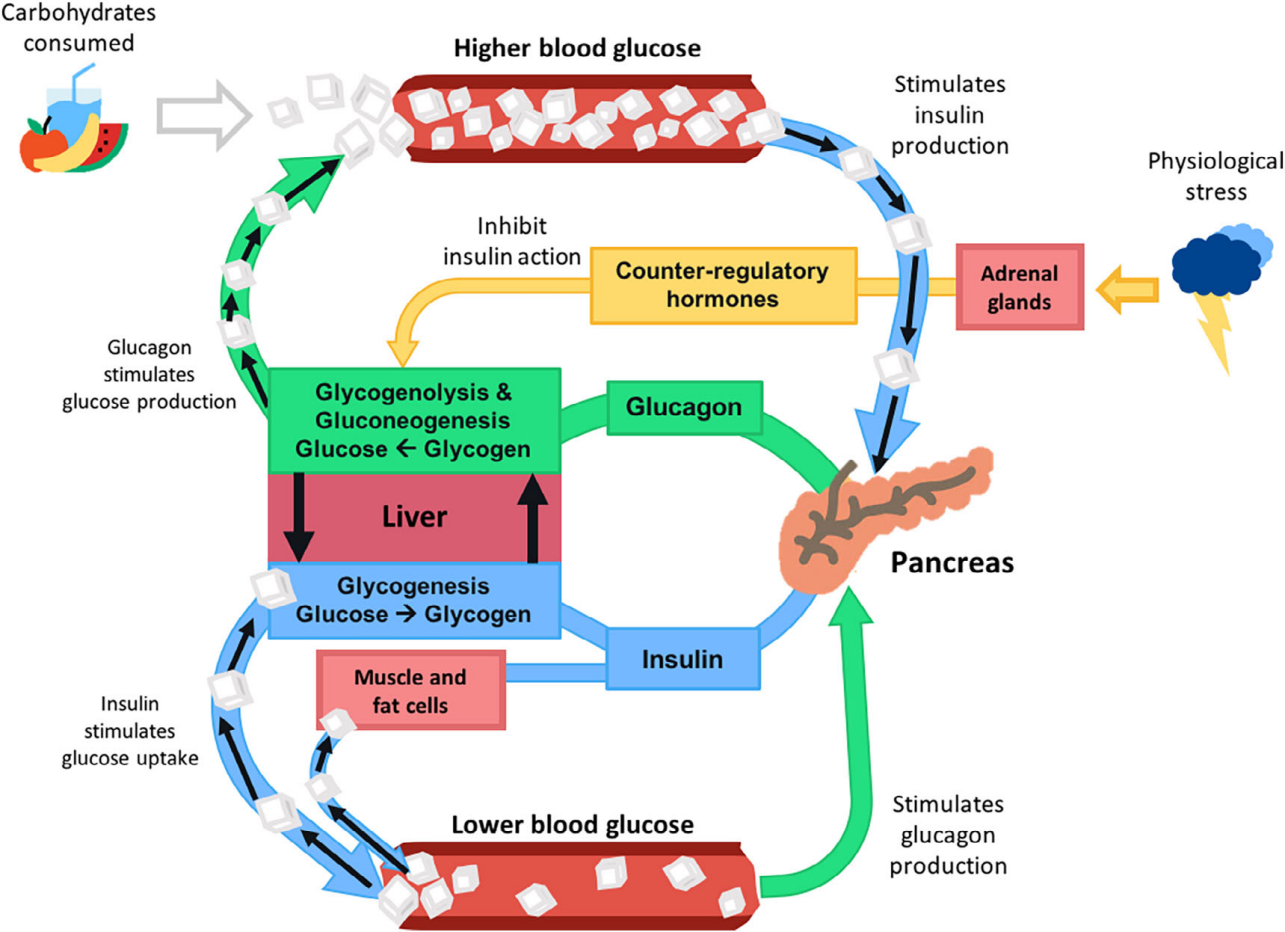
Primary aspects of glucose regulation in normal physiology. White cubes represent glucose molecules, blue connectors represent insulin pathways, and green connectors represent glucagon pathways. Yellow connectors distinguish counter‐regulatory hormones, such as cortisol, epinephrine, and norepinephrine. Figure redrawn from Holt, *Textbook of Diabetes*, 2010

This section provides a background for biomarkers and analytes that may be used in the context of T1D control. In particular, the functions and mechanisms of action for glucose, insulin, cortisol, lactate, BOHB, epinephrine, and alcohol are presented. Additionally, the physiological events that cause each to increase/decrease and their application in T1D management are summarized. Figure 1 illustrates the primary aspects of glucose regulation in normal physiology described in this section. Table 1 summarizes the feasible T1D analytes, and the physiological events that cause each to increase, with emphasis on those physiologic conditions related to T1D.

**TABLE 1:** Summary of physiological dynamics in individuals without T1D and the sensing technologies for reported analytes

Biomarkers	Physiological event(s) that cause(s) analyte to increase	Relation to T1D	Lab‐based sensing technology (biofluid)
Glucose	Meal intake	Primary indicator of hyper/hypoglycemia	Amperometry (capillary blood)
Insulin	Nutrients, including glucose, amino acids, and lipids	Absolute insulin deficiency in T1D	ELISA (blood/plasma/serum/saliva)
Glucagon	Low glucose levels, amino acids, exercise	Chronically elevated in T1D	Spectrophotometry and mass‐spectrometry (plasma)
Cortisol	Stress, fear, anxiety, pain, awakening, REM, illness, hypoglycemia	Elevates glucose levels	ELISA (plasma/serum/saliva)
Lactate	Exercise, severe illness (sepsis, hypotension)	Increasing during prolonged exercise	Amperometry (capillary blood)
Betahydroxybutyrate (BOHB)	Fasting, insulin deficiency	Markedly elevated with diabetic ketoacidosis (DKA)	Amperometry (capillary blood); spectrophotometry (venous blood)
Epinephrine/norepinephrine (adrenaline/noradrenaline)	Stress, "fight or flight," intense exercise, illness, hypoglycemia	Raises glucose, contributes to some symptoms of hypoglycemia	ELISA (blood/plasma/serum/urine)
Alcohol	Alcohol consumption	Increases risk of delayed hypoglycemia	Spectrophotometry (saliva/breath/sweat/urine)

### 2.1 Glucose

Blood glucose levels reflect the interaction of multiple regulatory pathways in the body, with a net balance between glucose production and uptake. Glucose production arises from three sources: (1) the dietary carbohydrates broken down and absorbed in the digestive system (i.e., meal intake), (2) glycogenolysis in liver, and (3) gluconeogenesis in the liver and kidneys. Glycogenolysis is the breakdown of glycogen, a storage form of carbohydrates. Gluconeogenesis, on the other hand, refers to the synthesis of glucose from noncarbohydrate precursors (e.g., amino acids, lipids). Glucose uptake occurs in most tissues in the body and can be insulin‐dependent (classically muscle and fat) or insulin‐independent (brain and most other tissues). Collectively, these aspects of glucose metabolism are regulated by the interaction of hormones including: (1) insulin, which stimulates glucose uptake and reduces glucose production; (2) glucagon, which inhibits glucose uptake and stimulates hepatic glucose production; and (3) other so‐called counterregulatory hormones such as cortisol, growth hormone, epinephrine, and norepinephrine, which also inhibit insulin action and increase blood glucose levels.^7^

### 2.2 Insulin

Insulin is a peptide hormone secreted by the beta cells within the pancreatic islets of Langerhans and is the dominant glucoregulatory hormone. In normal physiology, beta cells regulate the production of insulin in response to plasma levels of glucose, amino acids, keto acids, and fatty acids circulating within the plasma.^1^ Additionally, insulin secretion is stimulated by incretins, a group of metabolic hormones that are released from intestinal neuroendocrine cells in response to meals. Insulin is co‐secreted with amylin, which inhibits glucagon secretion, delays gastric emptying, and acts as a satiety agent. Thus, amylin can enhance some of the actions of insulin.

The role of insulin in glucose metabolism is to maintain normal blood glucose levels. Glucose uptake into muscle and adipose tissue is facilitated by insulin action, allowing glucose to enter into cells to be oxidized or stored as glycogen.^20^, ^21^ In the liver, insulin action causes a decrease in gluconeogenesis (generation of glucose) both directly, as well as indirectly by reducing release of precursor molecules such as amino and fatty acids from peripheral tissues, such as adipose and muscle.^22^ Insulin also causes an increase in glycogenesis (formation of glycogen from sugar). A large fraction of glucose absorbed from the small intestine is immediately taken up by hepatocytes (liver cells); insulin promotes storage of glucose as glycogen for later use.^20^

Insulin has important effects on lipid metabolism, stimulating lipogenesis, and inhibiting lipolysis. In the liver, stimulation of lipogenesis is particularly prominent after glycogen accumulates to high levels (roughly 5% of liver mass) and further glycogen synthesis is strongly suppressed. Under these conditions, insulin promotes synthesis of fatty acids from glucose, which can be stored in the liver or exported. Insulin also suppresses hepatic production of ketones from fatty acid substrates. In adipose tissue, insulin facilitates uptake of glucose, which can be used for synthesis of triglycerides.^20^ Insulin further regulates this process by inhibiting hormone‐sensitive lipase, leading to inhibition of lipolysis.^20^

Insulin also affects protein metabolism. Insulin stimulates protein synthesis via initiation and modulation of intracellular signal transduction pathways, which lead to increased amino acid uptake, and stimulation of mRNA translation, particularly when branched chain amino acids are available.^23^ Likewise, insulin signaling leads to potent inhibition of protein degradation (proteolysis). Finally, insulin increases the permeability of many cells to potassium, magnesium, and phosphate ions.^20^

In the case of T1D, insulin deficiency occurs due to an autoimmune destruction of pancreatic beta cells. The resulting abnormalities in carbohydrate, lipid, and protein metabolism of carbohydrates result in elevated levels of glucose in the blood, and if high enough, in the urine, as well as increased lipolysis, increased ketogenesis, and increased proteolysis. Thus, patients with T1D need exogenous insulin injections to not only keep blood glucose concentration in a safe range, but also to regulate these diverse aspects of cellular metabolism.

### 2.3 Glucagon

Glucagon is a peptide hormone produced in the alpha cells of the pancreatic islets.^24^ When glucose levels fall, secretion of glucagon increases. It acts on liver cells via glucagon receptors to promote glycogenolysis (breakdown of glycogen into glucose) and gluconeogenesis, resulting in a significant increase in blood glucose. Glucagon is used clinically to treat severe hypoglycemia; injection or nasal administration of pharmacologic doses of glucagon rapidly increases glucose levels within 10–15 min. There are also rare cases in which tumors produce excessive glucagon (glucagonoma) and cause hyperglycemia.

Glucagon has several effects on lipid metabolism; it stimulates adenylate cyclase, which in turn activates lipase, promoting lipolysis, or the breakdown of stored triglyceride into free fatty acids for catabolism by many tissues. Delivery of these fatty acids to the liver provides substrates for ketogenesis, also stimulated by glucagon action.^25^ Glucagon is also secreted during stress, such as exercise, and contributes to the maintenance of metabolic fuel homeostasis in these situations by helping to prevent hypoglycemia when glucose utilization by muscle is increased. Postprandial glucagon secretion also contributes to meal‐ending satiation.^26^ In the setting of T1D, plasma glucagon responses are abnormal; basal levels of glucagon can be high, but glucagon secretion in response to hypoglycemia is often inadequate, increasing the risk for severe hypoglycemia. An underlying cause of increased glucagon secretion may be related to the chronic effects of beta cell loss and insulin secretion, since severe beta cell loss removes a tonic restraint on the alpha cell. Elevated glucagon levels can also promote ketogenesis.^7^

### 2.4 Cortisol

Cortisol is a steroid hormone secreted by the adrenal gland in response to cues from the hypothalamus and pituitary gland—forming the hypothalamic–pituitary–adrenal (HPA) axis.^27^ Normal functioning of the HPA axis is required for health maintenance, and increased secretion of cortisol is required for appropriate adaptation to physiologic stress, such as illness. Cortisol levels exhibit diurnal regulation and are highest in the early morning, ranging from 8 to 19 μg/dl in the morning and from 3 to 10 μg/dl over the remainder of the day.^28^

Cortisol has particularly potent effects on glucose metabolism, causing a net increase in glucose levels by antagonizing insulin action (i.e., inducing insulin resistance), increasing hepatic gluconeogenesis, reducing glycogen synthesis, and decreasing glucose uptake in skeletal muscle.^29^ Cortisol secretion is also a key component of the “counterregulatory response” to hypoglycemia; if the cortisol response is inadequate, more severe or prolonged hypoglycemia may result.^30^ Moreover, high levels of cortisol increase glucagon levels and increase lipolysis in adipose tissue.

Psychological and physiological stress can cause an increase cortisol levels and can impair insulin sensitivity, with acute stress most severely affecting postprandial glucose levels.^31^ In individuals with T1D, increases in cortisol, as with acute illness, can result in not only increased glucose levels and increased insulin requirements, but also increased risk for ketoacidosis. Unfortunately, physiologic stressors (such as acute infection) may not be recognized, especially early in the course of illness. Thus, automatic detection of cortisol could help to predict and allow treatment to avoid increases in glucose arising from emotional and/or physical stress.

### 2.5 Lactate

Lactate is a three‐carbon metabolite constantly produced in the body during normal metabolism, with further increases during exercise. The concentration of blood lactate is typically 0.3–1.3 mM/L at rest, but can rise to greater than 20 mM/L during intense exercise depending on intensity. Individuals with T1D may have elevated lactate levels after exercise compared to controls.^32^ During anaerobic exercise, glycolytic metabolism yields pyruvate, which is converted to lactate by the enzyme lactate dehydrogenase.^5^ During progressive, incremental exercise in which the work rate is increased at regular intervals (typically, 1–4 min), blood lactate concentration increases gradually at first and then more rapidly as the exercise becomes more intense. A normal progressive, incremental exercise test elicits a peak of blood lactate concentration on the order of 9 mM at volitional exhaustion.^33^ Blood lactate concentration values may approach maximum values of 15–25 mM during the first few minutes following maximal effort of approximately 30–120 s of exercise duration. In the case of progressive, incremental exercise, peak values typically appear at approximately 3–8 min postexercise, but remain elevated for over 60 min.^34^, ^35^ Lactate levels increase to a greater extent during resistance exercise, given the predominance of glycolytic metabolism, than during longer‐term aerobic exercise, when oxidative metabolism dominates. Since lactate levels can serve as a marker of exercise, real‐time, automated measurement of lactate in individuals with T1D would be helpful to more precisely guide adjustments in delivery of insulin. Especially given that the glycemic response to exercise varies by type, intensity, and duration, which may be more specifically identified by changes in lactate levels.^36^

### 2.6 Ketones

The ketone bodies acetoacetate, BOHB, and acetone are derived from acetyl CoA produced via lipid oxidation. 3‐BOHB is formed from the reduction of acetoacetate in the mitochondria, while acetone is formed by spontaneous decarboxylation of acetoacetate.^25^ Under normal conditions, the serum concentration of ketone bodies is less than 0.3 mM/L. However, ketone synthesis increases during one of two cases: (1) when carbohydrate availability is reduced, such as with prolonged fasting, ketogenic diet, or exercise; or (2) when insulin levels are low, or counterregulatory hormones are increased, such as during severe illness. In these conditions, rates of lipolysis in adipose tissue are increased, leading to increased levels of free fatty acids, which are metabolized to ketones in the liver. These processes are further increased in the setting of insulin deficiency and increased counterregulatory hormones such as glucagon.

As with any metabolite, ketone clearance is a critical determinant of net plasma levels. Ketolysis is the process by which ketone bodies are metabolized in the mitochondria of many extrahepatic organs. Since ketone bodies are not bound to plasma proteins, they are freely filtered in the renal glomerulus. At very low plasma concentrations of ketone bodies, the urinary excretion of ketone bodies is less than 1%. However, when plasma levels increase beyond 0.1–0.2 mM, excretion increases and measurable amounts of ketone bodies appear in the urine.^37^

To reduce the urinary loss of ketones, the kidney is capable of reabsorbing a portion of the increased filtered load of ketoacids. Reabsorption of ketone bodies is saturable.^37^ Once the renal threshold is exceeded, plasma and urinary concentrations of acetoacetate are correlated. By contrast, rates of excretion of BOHB are exponential relative to blood levels.^38^ Insulin accelerates ketone body clearance, while insulin deficiency acts to decrease renal clearance of ketone bodies via unclear mechanisms.^25^

Ketogenesis may be considered an adaptive response during prolonged fasting, yielding a fuel that can be utilized by the brain for energy metabolism during conditions of low glucose availability. Transient and mild increases in ketones can also occur following sustained exercise, when fuel needs are increased and muscle blood flow is increased. However, during the postexercise period, ketone bodies may be generated as a result of increased mobilization of fatty acids, together with normalization of blood flow to the liver, yielding a transient rise in ketones by 30%.^39^, ^40^

However, with insulin deficiency, especially in the setting of dehydration, severe ketogenesis may result in increases in plasma levels by as much as 20‐fold, leading to reduction in pH—the combination of ketones and acid accumulation known as ketoacidosis. Diabetes‐related ketoacidosis (DKA) is a severe illness with high mortality.^40^If untreated, DKA can cause potentially fatal complications, such as cerebral edema, adult respiratory distress syndrome, and thromboembolic phenomena.^25^ The mortality rate of DKA has been declining in developed countries (less than 10%) but increasing in developing countries.^41^ Overall mortality from DKA in adults in the United States is less than 1%, but is greater than 5% in the elderly and those with life‐threatening illnesses.^2^ DKA is the leading cause of mortality among children. Cerebral edema occurs in 0.5%–1% of all episodes of DKA in children.^41^, ^42^, ^43^ This complication has a high mortality rate (21%–24%), and a substantial percentage of survivors (15%–26%) experience severe brain damage.^41^, ^42^, ^43^ Since complications of DKA are uncommon with prompt treatment, early diagnosis and treatment can circumvent morbidity and mortality.

Earlier references indicate that individuals with T1D who are treated with only short‐acting insulin (e.g., those on a pump) are particularly susceptible, as interruption of insulin delivery for even a short period of time (a few hours) can rapidly initiate ketogenesis and DKA^44^, ^45^; however, more recent publications do not reveal similar increased DKA rates with insulin pump therapy.^46^ Thus, continuous or frequent measurements of ketone levels, particularly BOHB, may be helpful for the prevention, early detection, and initiation of treatment for DKA in the management of intercurrent illness, as surveillance for adequacy of insulin infusion in those who are pump‐treated, during and after exercise, and in other settings.

### 2.7 Epinephrine (adrenaline) and norepinephrine (noradrenaline)

Epinephrine is the primary catecholamine produced by the central portion of the adrenal gland (the adrenal medulla) in response to activation of the sympathetic nervous system.^47^ The adrenal medulla itself is composed of chromaffin cells, which are modified postganglionic sympathetic neurons. These cells receive input from presympathetic neurons in the rostral medulla of the brain, which are, in turn, under the control of central and/or peripheral glucose‐sensing neurons.^48^ Epinephrine is released into the bloodstream and can act at distant sites via alpha‐ and beta‐adrenergic receptors on cell surfaces. In contrast to many other hormones, the action of catecholamines is rapid and relatively transient in nature.

Epinephrine plays a particularly important role as a major mediator of the acute counter‐regulatory response to acute reductions in plasma glucose levels. Epinephrine acts directly to promote glycogenolysis and gluconeogenesis in the liver and to reduce glucose uptake by muscle. Although its effect on glycogenolysis rapidly wanes, effects on gluconeogenesis and glucose disposal persist even after the exercise or stress period has ceased.^49^ Collectively, these effects rapidly restore circulating glucose levels and contribute to the “flight or fight” response. During acute exercise, epinephrine levels increase by 1.5 to over 20 times basal concentrations, with magnitude of increase related to duration and intensity. For a given duration, the circulating epinephrine concentrations increase exponentially with the intensity of exercise.^50^ Moreover, endurance‐trained individuals have a higher capacity to secrete adrenaline than untrained individuals. The values measured immediately before exercise were significantly higher in trained compared with untrained individuals, suggesting that the trained individuals developed anticipating capacities.^51^ Intensity of exercise also determines the time course of normalization of epinephrine levels; after brief and intense exercise, plasma epinephrine concentrations quickly return to their baseline values and may even decrease by 35% after 1 min of rest.^52^, ^53^

Epinephrine is particularly important for counterregulatory responses to hypoglycemia in individuals with T1D as the glucagon response to hypoglycemia is typically lost within 5 years of diagnosis of T1D.^54^ Unfortunately, repeated events of hypoglycemia can also lead to reduced epinephrine secretion and “hypoglycemia unawareness,” in which the symptoms of hypoglycemia are no longer perceived, resulting in unexpected onset of severe neuroglycopenia, or cognitive dysfunction, which may result in falls, loss of consciousness, or seizures.^55^

On receiving distress signals from the amygdala, the adrenal medulla also releases norepinephrine, in addition to epinephrine, into the blood stream. While norepinephrine is stored only in small amounts in adrenal tissue, its major site of storage are the neurons of the sympathetic nervous system.^56^ Thus, norepinephrine, also called noradrenaline, functions mainly as a neurotransmitter in the central nervous system and sympathetic nervous system. Norepinephrine effects are mediated by the family of adrenergic receptors and aim to transmit the “fight or flight” response, as well as stimulate alpha‐adrenoreceptors, which are situated in the arteries. Since the target cell determines the cellular effect, the action of norepinephrine produces a different effect in each tissue.^56^ Along with epinephrine, norepinephrine increases heart rate, blood glucose levels by triggering the release of glucose from energy stores, and the levels of circulating free fatty acids by aiding fat breakdown. However, unlike epinephrine, norepinephrine increases blood pressure, promoting vasoconstriction. Norepinephrine activity is mainly terminated through inactivation by reuptake into nerve endings, which protect it from metabolism until it is released following nerve stimulation.^56^

### 2.8 Alcohol

When alcohol (ethanol) is consumed, it is readily absorbed from the gastrointestinal tract. The breakdown of alcohol involves two enzymes—alcohol dehydrogenase and aldehyde dehydrogenase—that allow its elimination from the body. Alcohol dehydrogenase catalyzes the oxidative metabolism of alcohol into a highly toxic compound called acetaldehyde, which is generally short‐lived.^57^ Subsequently, within in the liver, acetaldehyde is oxidized to acetate, which is a less toxic substance.

Alcohol influences glucose metabolism in several ways in individuals with T1D. First, alcohol inhibits gluconeogenesis, thus reducing the liver's ability to release glucose into the blood. Alcohol also increases glycogen phosphorylase activity, depleting hepatic glycogen stores. Since alcohol inhibits both gluconeogenesis and reduces glycogen stores, intake of large quantities of alcohol may provoke hypoglycemia; this process may be aggravated if food intake has been inadequate. Effects of alcohol may be delayed, with increased risk of hypoglycemia as long as 24–48 h after consumption. This delay may be due in part to the reduction in nocturnal growth hormone secretion after alcohol consumption.^58^, ^59^, ^60^ Moreover, after alcohol is metabolized, hepatic insulin sensitivity is increased, leading to the restoration of glycogen stores and reduction in blood glucose levels.^61^ Ethanol metabolism also reduces the oxidation of lactate to pyruvate, potentially increasing accumulation of lactate, as in the case of prolonged ethanol consumption and subsequent alcoholic lactic acidosis. Monitoring alcohol concentrations in real time may allow improved insulin delivery protocols to reduce delayed hypoglycemia.

## 3 DETECTION TECHNIQUES

**FIGURE 2:**
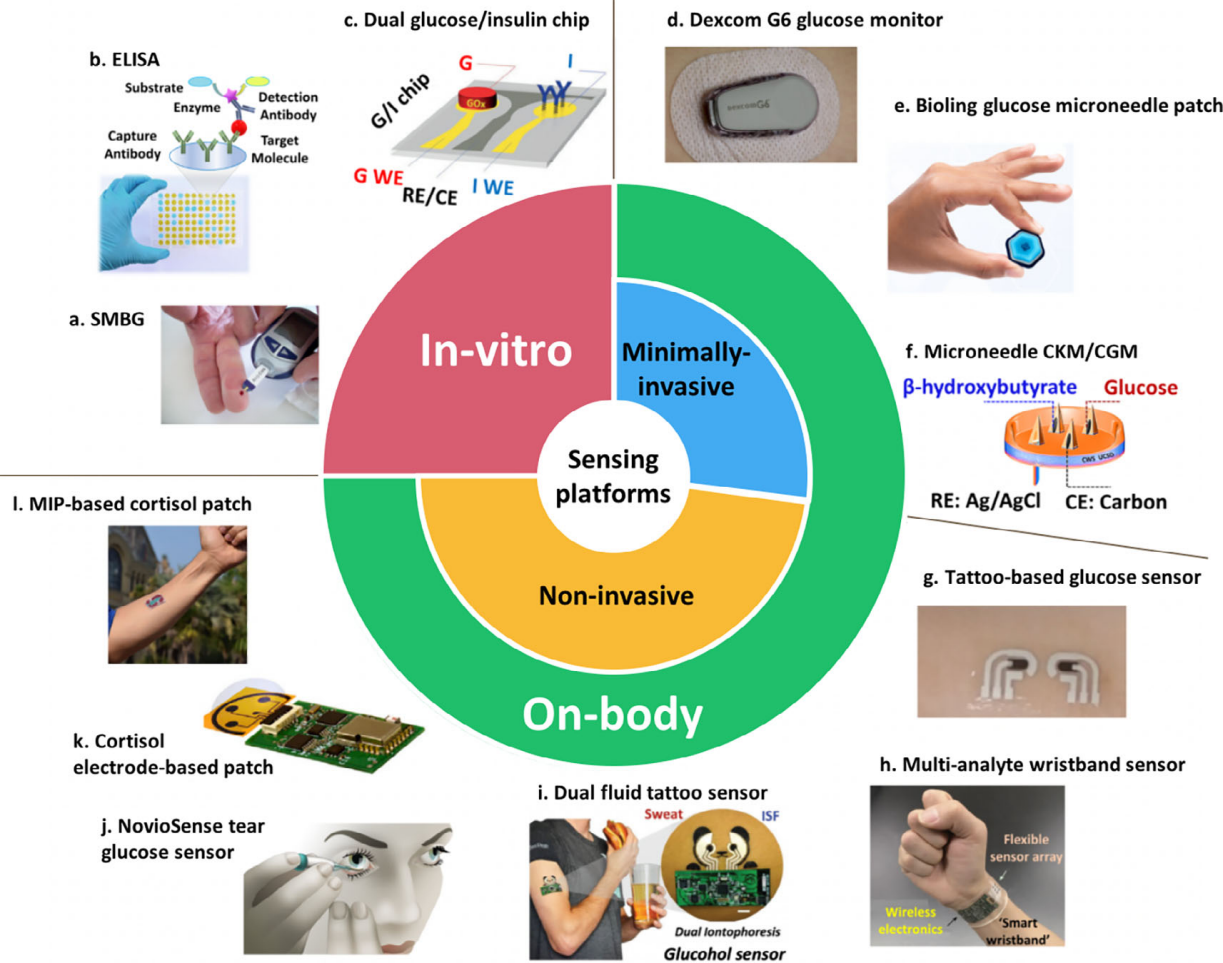
Representative examples of different in‐vitro and on‐body sensing approaches to measure diabetes‐related biomarkers. (a) Self‐monitoring blood glucose (SMBG) meter. Reprinted by permission from Reference 112. (b) Schematic illustration of the working principle of enzyme‐linked immunosorbent assay (ELISA) method for analyzing protein biomarkers. (c) Dual‐analyte glucose‐insulin (G/I) detection chip. Copyright (2019) Wiley. Used with permission from Reference 70, John Wiley and Sons. (d) Free CGM, Dexcom G6. Reproduced by permission from Dexcom,^75^ Copyright (2020). (e) Microneedle‐based glucose monitoring system by Biolinq. Reprinted by permission from Biolinq,^84^ Copyright (2020). (f) Microneedle‐based CKM coupled with CGM. Reprinted from American Chemical Society,^85^ Copyright (2020). (g) Tattoo‐based noninvasive glucose monitoring. Reprinted by permission from American Chemical Society,^91^ Copyright (2020). Further permissions related to the material excerpted should be directed to the ACS. (h) Fully‐integrated wristband sensor consisting of glucose, lactate, sodium, potassium, and temperature sensors. Reprinted by permission from Springer Nature: Nature,^98^ Copyright (2016). (i) Epidermal tattoo‐based patch for simultaneous measurement of interstitial fluid (ISF) glucose and sweat alcohol. Reprinted from John Wiley and Sons,^99^ Copyright (2018). (j) NovioSense tear glucose sensor. Reprinted by permission from References 105, 113. Further permissions related to the material excerpted should be directed to the ACS. (k) Cortisol sensor patch based on laser‐engraved graphene electrodes for analyzing cortisol in sweat. Reprinted from Reference 106, Copyright (2020), with permission from Elsevier. (l) Molecularly imprinted polymer (MIP) recognition‐based cortisol patch for analyzing cortisol in sweat. Reprinted from Reference 107. Copyright The Authors, some rights reserved; exclusive licensee American Association for the Advancement of Science. Distributed under a Creative Commons Attribution NonCommercial License 4.0 (CC BY‐NC). http://creativecommons.org/licenses/by‐nc/4.0/

Given the importance of the metabolites described in healthy metabolism (Section 2), the ability to detect metabolites—beyond glucose—in real‐time would provide key information to guide more physiological and safe treatment for T1D. This section provides an in‐depth description of the major scientific and technological advances in the detection of diabetes‐related biomarkers. Detection devices available for different biomarkers are discussed in terms of invasiveness to provide the reader with a clear yet concise picture of the previous and current trends into this evolving field. While in‐vitro chips have long been available, the recent advances in biosensor technology have stimulated extensive efforts toward developing on‐body minimally and noninvasive continuous monitoring. These efforts follow dynamic concentration changes and bridge the gap between laboratory and clinical POC detection strategies. Figure 2 provides the most representative examples in different fields of POC and on‐body wearables.

### 3.1 In‐vitro chips

The technology of blood glucometers has come a long way since the introduction of the first electrochemical glucose meter by Medisense in 1987.^62^ The current generation of self‐monitoring blood glucose (SMBG) strips provide fast (5‐s) and accurate measurements using ultra‐small volumes of blood samples (as low as 200 nl), as well as connectivity to smartphones, which enable users to easily share glucose data with their caregivers and family members (Figure 2(a)).^19^ Representative examples of recently introduced blood glucose meters are the Sanofi's iBGStar, which is the first iPhone glucose meter,^63^ the Dario's “all‐in‐one” pocket‐sized glucose meter,^64^ and Sentec's No‐strip cassette‐based technology.^65^ Such test strips rely on glucose oxidation through a mediated reaction with an immobilized enzyme (glucose oxidase or dehydrogenase) and measure the resultant amperometric current of the mediator oxidation reaction. Ferricyanide, Os^2+/3+^, and ferrocene derivatives are the most commonly used mediators.^18^ In addition to glucose, amperometric test strips have been developed to measure blood levels of lactic acid and the ketone body BOHB.^66^ However, the repetitive use of finger pricking‐based SMBG can be painful and does not provide a continuous temporal profile of glucose or other metabolites' levels.^67^ Alternative approaches are also under development, including test strips that can measure glucose from external biofluids other than blood. For example, iQ Group Global in Australia is currently working on disposable saliva glucose strips, which rely on Organic Thin Film Transistor technology.

Unlike enzyme‐based strips for on‐the‐spot measurements of metabolite diabetes biomarkers, laboratory‐based analysis of T1D‐based analytes, such as insulin, cortisol, C‐peptide (a marker of endogenous insulin production), and insulin antibodies (present in most people who receive exogenous insulin; such antibodies can lead to higher circulating insulin levels). These markers commonly rely on enzyme‐linked immunosorbent assay (ELISA) kits due to their analytical sensitivity and commercial availability (Figure 2(b)).^68^ However, since these immunoassays are usually performed in centralized laboratories using serum or plasma samples, results are not available immediately. Additionally, trained personnel are needed to obtain sufficient amounts of serum/plasma, often from milliliter quantities of venous blood, and to perform the relevant assays, which hinders the application of these kits for self‐monitoring purposes. Therefore, self‐monitoring strip‐based devices for rapid decentralized testing of these analytes using a single microliter drop of capillary blood would be a major leap forward in diabetes care.

Self‐monitoring chip‐based POC devices for rapid decentralized testing of analytes using a single microliter drop of blood would be a major leap forward in diabetes care and may be used for the simultaneous detection of multiple analytes (e.g., glucose, insulin, and cortisol).

An attractive label‐free approach to measure insulin in undiluted serum samples has been reported by Davis' group, which was based on the non‐Faradaic electrochemical impedance spectroscopy.^69^ A notable advancement in multiplexed detection of T1D markers has recently been made toward simultaneous amperometric detection of glucose and insulin in the same microliter sample droplet, for example, undiluted saliva and capillary blood samples, by combining enzymatic and immunochemical assays on a single microchip (Figure 2(c)).^70^ Even more recently, a dual microchip was reported for simultaneous detection of insulin and cortisol through the integration of different enzymatically‐tagged sandwich and competitive immunoassay formats.^71^ Such multiplexed assays, taking less than 25 min and using small microliter droplets of untreated blood, hold considerable promise toward improved estimation of insulin sensitivity and enhanced regulation of glucose levels. These promising examples require further clinical validation for widespread decentralized testing of T1D‐based protein biomarkers. Table 2 summarizes the features of invasive in‐vitro sensing devices.

**TABLE 2:** Performance characteristics of invasive in‐vitro sensing devices

Concept (analytes)	Features	Description	References
*Blood meters*; Enzyme‐based (glucose; BOHB; lactate)	Measurement technique	Electrochemical (amperometric)	[66]
	Body fluid	Capillary blood	
	Frequency	Any time required; readout in 5 s for glucose and 10 s for BOHB	
	Advantages	Fast, cost‐effective, and portable; easy fabrication; high precision and accuracy	
	Disadvantages	Invasive sampling; single analyte detection; incapability to continuously monitor the biomarkers	
	Scalability	High (established screen‐printing technology)	
	Utilization	Clinical	
*ELISA kit*; Bioaffinity‐based (cortisol, insulin, C‐peptide, insulin antibodies)	Measurement technique	Optical (colorimetric)	[68]
	Body fluid	Plasma, serum	
	Frequency	Depending on centralized lab analysis	
	Advantages	High analytical sensitivity and commercial availability of kits; well‐established analysis protocols in clinical practice	
	Disadvantages	Long analysis times; long delay times between sampling and analysis; samples pretreatment and/or dilution; expensive equipment; not adaptable to use by patient; single analyte detection	
	Scalability	High	
	Utilization	Clinical	
*G/I biochip*; Hybrid enzyme/bioaffinity‐based (glucose; insulin)	Measurement technique	Electrochemical (amperometric)	[70]
	Body fluid	Capillary blood	
	Frequency	Any time required; simultaneous detection in less than 25 min	
	Advantages	Multiplexed, simultaneous analysis; speed; no need to sample pretreatment; low required sample volumes; can be expanded to measuring other analytes; low cost	
	Disadvantages	Invasive; not adaptable to continuous monitoring	
	Scalability	High (lithography‐free masking/sputtering fabrication)	
	Utilization	Laboratory	

### 3.2 On‐body detection

Wearable devices have recently emerged as the next‐generation technological platforms to address the challenges of in‐vitro detection systems. These body‐worn platforms are capable of providing real‐time analytical information by continuously measuring the concentrations of various biomarkers in minimally‐ or noninvasive fashion. CGM technology, relying on minimally‐invasive ISF glucose tracking, has already gained tremendous commercial success. However, major efforts are ongoing toward developing noninvasive devices, which can offer simultaneous measurements of other diabetes‐related markers along with glucose. Here, the major scientific and technological advances in the arena of on‐body wearables for detecting diabetes‐related markers are summarized. Tables 3 and 4 provide on‐body minimally‐ and noninvasive detection approaches, respectively, in terms of measurement frequency, advantages, disadvantages, scalability, and utilization (laboratory vs. clinical).

**TABLE 3:** Performance characteristics of on‐body minimally invasive devices based on representative examples

Concept (analytes)	Features	Description	References
*CGM devices*; Enzyme‐based (glucose)	Measurement technique	Electrochemical (amperometric)	[74, 75]
	Body fluid	ISF	
	Frequency	Every 5 min	
	Advantages	Continuous monitoring; calibration‐free; predictive alarms for high/low glucose levels; adaptable to closed‐loop feedback controlling	
	Disadvantages	High cost; limited lifetime; single analyte detection (glucose)	
	Scalability	High	
	Utilization	Clinical	
*Microneedle‐based sensor*; Enzyme‐based (glucose)	Measurement technique	Electrochemical (amperometry)	[84]
	Body fluid	ISF	
	Frequency	—	
	Advantages	Adaptable to multiplexed detection; Much less painful than current CGM devices	
	Disadvantages	More complex fabrication than current CGM devices	
	Scalability	Low	
	Utilization	Clinical evaluation stage	

**TABLE 4:** Performance characteristics of on‐body noninvasive devices based on representative examples

Concept (analytes)	Features	Description	References
*Tattoo‐based sensor*; Enzyme‐based (glucose; lactate; alcohol)	Measurement technique	Electrochemical (amperometric)	[91, 95, 96, 99]
	Body fluid	ISF; Sweat	
	Frequency	Real‐time	
	Advantages	Noninvasive; low cost; continuous sampling	
	Disadvantages	Glucose dilution during extraction; nonuniform and low extraction rates; contamination by other sources	
	Scalability	High	
	Utilization	Laboratory	
*Wristband*; Hybrid enzyme/ionophore‐based (glucose; lactate; Na^+^; K^+^)	Measurement technique	Electrochemical transistor	[98]
	Body fluid	Sweat	
	Frequency	Real‐time	
	Advantages	Multiplexed metabolites detection; fully‐integrated device	
	Disadvantages	Contamination by skin or old sweat; low extraction rates; sweat is less accepted fluid than ISF; exercise‐based sampling	
	Scalability	Low	
	Utilization	Laboratory	
*Cortisol sensor patch*; MIP‐based (cortisol)	Measurement technique	Electrochemical transistor	[103]
	Body fluid	Sweat	
	Frequency	—	
	Advantages	Noninvasive; higher stability than immune‐based assays	
	Disadvantages	Regeneration; selectivity and reproducibility of the device; sweat is less accepted body fluid than ISF; single analyte detection	
	Scalability	Low	
	Utilization	Laboratory	
*Ophthalmic insert in lower eyelid*; Enzyme‐based (glucose)	Measurement technique	Electrochemical (amperometry)	[105]
	Body fluid	Tear	
	Frequency	Real‐time	
	Advantages	Noninvasive	
	Disadvantages	Limited lifetime (first generation devices are planned to work for 2 weeks); sweat is less accepted body fluid than ISF	
	Scalability	High	
	Utilization	Clinical evaluation stage	

#### 3.2.1 Minimally‐invasive devices

The current CGM technology from four leading companies, Dexcom, Abbott, Medtronic, and Eversense, relies on needle‐based enzyme electrodes. This approach is becoming the new standard of care for many with T1D because it can address key drawbacks of in‐vitro blood glucose strips. Such wearable, on‐body electrochemical sensor platforms offer continuous accurate glucose readings for 7‐ to 14‐day periods with mean absolute relative difference values approaching those of blood glucose meters (10%). As the sensor component sits in the subcutaneous fat tissue to measure the interstitial glucose, these commercial CGM devices are mainly accepted as minimally invasive.^72^, ^73^ Abbott's FreeStyle Libre Flash, FDA‐cleared in July 2018, has an extended sensor lifetime of 14 days, is calibration‐free, and provides intermittent glucose levels by scanning a skin‐worn patch with a hand‐held scanner device.^74^ The Dexcom G6, FDA‐approved in March 2018, offers a wear time of 10 days without the need for fingerstick calibrations and has an alert to notify if glucose levels fall rapidly or reach a prespecified low glucose threshold (e.g., less than 55 mg/dl; Figure 2(d)).^75^ Finally, Medtronic's Guardian Sensor 3, with a lifetime of up to 7 days, is a part of the Medtronic MiniMed^TM^ 670G device, which was released in 2017 as the first hybrid closed‐loop system that automatically regulates basal insulin delivery based on glucose readings.^76^ CGM devices are the first, and currently only, type of sensor to fall under the FDA's integrated continuous glucose monitor designation in that they can communicate with insulin pumps. Despite these key achievements in the CGM arena, further improvements to address their limited long‐term stability, high cost, and relatively large size remain. As further miniaturization ensues, improved cost‐effectiveness, guaranteed data security, improved accuracy and precision, as well as more advanced software and device features are needed.

CGM sensors are subcutaneously inserted under the skin and can remain in place up to 7–14 days, potentially as long as 90 days, providing 288 daily measurements. They are currently the only type of sensor to fall under the FDA's integrated CGM designation and can communicate with insulin pumps via Bluetooth.

CGM sensors are minimally invasive. After insertion, a subcutaneous electrode remains under the skin and, depending on the device, can stay in place for up to 14 days,^11^ potentially for as long as 90 days when using the Sensionics Eversense® CGM, as opposed to capillary fingerstick measurements, which are often repeated multiple times per day. The frequency of traditional home blood glucose monitoring varies by age and generally ranges from 4 to 7 times daily, and each measurement requires a separate finger prick.^4^, ^77^, ^78^ Of note, this frequency of BG monitoring actually reflects the time before substantial market penetration of CGM devices, which has led to reduced BG monitoring. As a result, user compliance becomes an important factor that can compromise the true real‐time application of SMBG. This approach contrasts with the 288 daily measurements obtained with the use of CGM, which only requires insertion of a cannula once every 7, 10, or 14 days, depending on the CGM device. Therefore, the overwhelming majority of glucose readings are obtained noninvasively and painlessly. Alternative approaches include the Sensionics Eversense® CGM, in which an 18.3 × 3.5 mm sensor that lasts up to 90 days is implanted under the skin, and then only a sticker patch (the transmitter) is required to be placed on the skin to transmit the CGM readings to a receiver or app on a mobile device.

Current commercial CGM devices have improved accuracy, with mean absolute relative differences of only ~9% compared to gold standard measurements.^79^ However, due to the size of the CGM transmitter that the sensor connects to, an adhesive must be used to hold the device to the skin. Allergic contact dermatitis to the adhesive, in particular the adhesive colophonium, has been reported to be fairly common, with over half of individuals experiencing some form of skin reaction in some reports.^80^ It should be noted that infection from CGM cannulas are extremely uncommon.

Wearable microneedle sensors can reach the ISF in less than half a millimeter (CGM devices require 5–11 mm to access the subcutaneous fat layer) and can accommodate multianalyte sensors on a single miniaturized sensor array path (e.g., glucose, BOHB, and ketones).

Within the last few years, microneedle‐based wearable sensors have received tremendous attention for ISF glucose monitoring.^81^, ^82^ These sensors reach the ISF in less than half a millimeter, in contrast with the current CGM devices, which access the subcutaneous fat layer 5–11 mm under the skin, and cover less surface area.^83^ Some startup companies, such as Biolinq (San Diego, CA), are developing microneedle technology as a painless alternative to the state‐of‐the‐art CGM devices (Figure 2(e)).^84^ Another advantage of microneedle devices is their capability to accommodate multianalyte sensors on a single miniaturized sensor array patch. The first example of a continuous ketone body monitoring (CKM) system for the measurement of ISF BOHB levels has recently been described by Teymourian et al., which will be integrated with glucose and lactate microneedle biosensors to achieve a multiplexed minimally‐invasive diabetes monitoring patch (Figure 2(f)).^85^ Mohan et al., have demonstrated microneedle‐based real‐time alcohol monitoring.^86^ Although such microneedle‐based approaches are still in the clinical evaluation stage, they are expected to play a major role in the future diabetes market owing to their distinct multiplexed sensing capability. Additionally, the possibility of integrating sensing and delivery components on a single microneedle array will open unprecedented opportunities toward realizing a minimally‐invasive, fully‐automated closed‐loop device for people with diabetes.^87^ However, efficient sterilization strategies should be adopted to ensure the safe deployment of these microneedles, as well as any other platform that is in direct contact with the tissues inside the body, to avoid any possibility of infection. For example, gamma ray radiation is a versatile method that has been used for the sterilization of microneedle arrays before on‐body analysis.^88^

“…integrating sensing and delivery components on a single microneedle array will open unprecedented opportunities toward realizing a minimally‐invasive, fully‐automated closed‐loop device for people with diabetes.”

#### 3.2.2 Noninvasive wearable sensing systems

With the rapid progress in materials science and fabrication techniques, as well as the emergence of smartphones and other mobile devices, substantial efforts have been aimed at establishing wearable noninvasive devices. These devices are designed to avoid breaking the skin's integrity and will enable the monitoring of biochemical markers in peripheral body fluids, such as tears, sweat, and saliva, or in the ISF.^89^

Reverse iontophoresis (RI) is currently the most practical technique in developing biosensors that do not break the skin's integrity, relying on a mild electrical current to the epidermis. This technique has been combined with flexible, skin‐worn printable temporary tattoos for continuous ISF glucose measurement, as well as integrated with a flexible paper battery with a gold electrode for ISF extraction.

The most practical technique in developing noninvasive biosensors has been through RI, which relies on applying a mild electrical current to the epidermis, causing an electro‐osmotic flow of the ISF across the skin. Inspired by the early development of the GlucoWatch glucose‐monitoring biographer,^90^ this technique has recently been combined with flexible, skin‐worn printable temporary tattoos to continuously measure glucose extracted from the ISF in connection to a glucose oxidase‐Prussian Blue transducer (Figure 2(g)).^91^ Other promising examples of RI‐based glucose monitoring patches include the integration of a flexible paper battery with a gold electrode for enhanced ISF collection^92^ and an array of miniature pixels containing Pt‐decorated graphene working electrodes.^93^ This technology allows ISF extraction through individual, privileged follicular pathways across the skin. Clinical translation and potential commercialization of such attractive biosensing systems will require critical large‐scale validation over long periods. RI‐based ISF sampling technology has already been designed by Nemaura Medical Inc., which is developing SugarBEAT as the first noninvasive CGM device offering glucose readings every 5 min.^94^

Sweat has also shown great promise for noninvasive monitoring of various T1D markers, such as lactate, alcohol, and glucose.^95^, ^96^, ^97^ This approach can either be based on excreted sweat during physical exercise or by iontophoretic sweat stimulation.^95^, ^96^, ^97^ A notable advancement was illustrated by Gao et al., who developed a fully integrated flexible wrist‐band sensor array for multiplexed measuring of glucose, lactate, sodium, potassium, and skin temperature (Figure 2(h)).^98^ In another promising work, the authors presented dual biofluid sampling and analysis on a single wearable epidermal platform, enabling simultaneous on‐body detection of sweat‐based alcohol and ISF glucose in human subjects (Figure 2(i)).^99^ In addition, the combination of a glucose measuring patch with a thermo‐responsive microneedle has been shown to be useful for closed‐loop feedback therapy using sweat biofluid.^100^

Wearable bioaffinity platforms enable POC measurements for protein markers, such as cortisol and insulin, but cannot yet provide continuous real‐time monitoring.

Tears represent another bodily fluid that can be exploited in noninvasive monitoring of chemical markers. In this regard, contact lens‐based systems offer significant promise as they can be worn without irritation and are in direct contact with the ocular fluid.^101^ Representative examples are graphene‐based wearable multifunctional sensors that take advantage of hybrid structures of 1D and 2D nanomaterials for wireless detection of tear glucose and intraocular pressure,^102^ as well as a miniature self‐powered glucose sensor, capable of harvesting electrical power from basal tears.^103^ Another development has been reported based on a wireless contact lens that is able to continuously measure glucose and deliver drug therapies on demand to treat diabetic retinopathy.^104^ NovioSense (Nijmegen, the Netherlands) is developing a noninvasive tear glucose sensor for T1D that relies on a small spring‐like device placed in the lower eyelid. The company recently announced the completion of a phase II clinical trial in 24 participants with T1D (Figure 2(j)).^105^

Artificial receptors, such as molecularly imprinted polymers (MIPs) and aptamers, can offer viable solutions toward continuous on‐body monitoring of protein biomarkers, but still require addressing key associated challenges before use in future applications.

While there are several recent examples of on‐body noninvasive metabolite monitoring devices that rely on enzymatic biorecognition events, the development of wearable bioaffinity platforms for continuously measuring protein markers, such as cortisol and insulin, is a challenging task. Major challenges reflect inherent problems of affinity‐based sensing systems, including additional incubation and washing steps, slow recognition, strong irreversible binding events, and significantly lower analyte concentrations in bodily fluids. A recent study described an integrated wireless sensing device for detecting in‐vitro cortisol in sweat and saliva samples (Figure 2(k)).^106^ The cortisol sensor, relying on a competitive immunoassay format on a laser‐induced graphene electrode, enabled a wearable and POC sweat analysis. However, such a system cannot provide continuous real‐time monitoring of cortisol levels. Artificial receptors, such as MIPs and aptamers, can offer viable solutions toward continuous on‐body monitoring of protein biomarkers. An attractive example relies on the use of an electrochemical transistor integrated with a tailor‐made biomimetic polymer layer acting as the molecular memory layer to detect sweat cortisol (Figure 2(l)).^107^ Despite the innovative strategy that has been applied, future applications of such MIP‐based systems require addressing the key associated challenges, including their low reproducibility and selectivity compared to their immune‐based counterparts. Conformationally‐changing aptamer receptors can be more attractive for future on‐body monitoring of protein markers, including T1D‐related cortisol and insulin hormones, although such systems have so far been solely reported for in‐vivo monitoring of small molecules. The expansion toward monitoring larger biomolecules will require a multidisciplinary effort. Overall, current efforts in the arena of noninvasive wearables are expected to lead to multiplexed monitoring systems capable of simultaneous measurements of multiple biomarkers in real‐time for enhanced T1D management.

### 3.3 Summary of biomarkers and detection techniques

In summary, ISF glucose sensing via minimally‐invasive CGM devices is quickly gaining traction and increased acceptability by both patients and clinicians. With accuracies matching those of SMBG strips, on‐body CGM platforms provide calibration‐free, continuous monitoring capability. Microneedles are rapidly emerging platforms that can offer less invasiveness and multiplexed sensing advantages compared to CGMs, while maintaining the same reliability, and are thus expected to appear in the market in the near future. Microneedles can expand the range of detectable analytes to other metabolites, including BOHB, lactate, and alcohol, using the same enzyme‐based protocols. For other biomarkers such as insulin, cortisol, and glucagon, the assays are currently limited to centralized labs; major efforts are currently directed toward developing self‐monitoring strip‐based meters, which can reliably measure these biomarkers within an ultrasmall drop of capillary blood in home and POC settings.

## 4 BIOMARKER ANALYTES AND GLYCEMIC CONTROL

While AID and manual insulin delivery systems have reached their peak in terms of performance, the inclusion of clinically relevant analytes and detection techniques could markedly improve diabetes management, as well as reduce T1D‐related complications. The paramount drive for closed‐loop AID and AP systems is to improve glucose time in range; glucose levels should remain within a safe (euglycemic) range, between 70 and 140 mg/dl. These systems rely solely on inputs from glucose signals. The delay in the metabolic impact of insulin injections, exercise, alcohol consumption, and other physiologic effects are not immediately reflected in glucose levels, and as a result, rapid response to glucose variability due to over‐ or under‐calculation of insulin dosing is not available. Introducing advances in sensor technology would provide additional information that could be used as feedback for insulin delivery systems, and for enhanced control of AP systems, which can improve T1D management.

Several scenarios, including delayed effects of insulin, stress, exercise, DKA, and alcohol consumption have been identified as particular challenges in T1D treatment. From a control perspective, the regulated variable is glucose. While the relationship between glucose and insulin can be estimated, there is still no method of measuring insulin apart from laboratory‐based techniques. Currently, insulin on board (IOB) is typically determined through the approximation of insulin decay curves. This approach provides only a rudimentary approximation of IOB, but provides no actual insulin level values. The ability to monitor insulin within a home environment, such as through POC devices, may alleviate over deliveries, as well as improve the safety of AID.^108^

Exercise is a widely tested challenge in T1D. Both during exercise and in early and late recovery, there is increased insulin sensitivity.^109^ As reported by Chu et al. and Tamborlane, children with T1D who are on fixed insulin doses are at “triple jeopardy” for hypoglycemia on nights following exercise, potentially due to: (1) peripheral glucose utilization increasing during recovery; (2) counterregulatory hormone responses impaired by sleep; and (3) unchanged insulin concentrations because of a fixed basal insulin treatment regimen.^109^, ^110^ Measurements of lactate and epinephrine levels may allow for the detection of unannounced exercise, particularly in individuals with preserved epinephrine secretion. Additionally, measurements of norepinephrine may allow for early detection of hyperglycemic events, as well as conditions of distress. Monitoring these analytes may also provide insight into detection and prediction of insulin sensitivity, which would significantly enhance the safety of AP systems. Ultimately, these additional inputs may allow AP control algorithms to rapidly detect and respond to physiologic stress including physical activity, mitigating dysglycemic events.

## 5 CONCLUSIONS

By leveraging the remarkable advances in wearable sensor technology and introducing additional analyte signals obtained via in‐vitro and on‐body (minimally and/or noninvasive) biosensors, the physiological function of a healthy pancreas may be more effectively bio‐mimicked, ultimately improving glycemic control. In particular, integrating measurements of insulin, cortisol, lactate, ketones such as BOHB, epinephrine, and alcohol may aid in the glycemic control and management of T1D. While some of these analytes can be obtained within a laboratory setting, measurements for all via immunoassays cannot yet be used in ambulatory settings.^70^ However, recent technological advances in wearable sensor capability may soon allow for on‐demand information, similar to SMBG meters.^16^, ^17^, ^18^, ^19^, ^70^ These sensors have the potential to lead to POC measurements, advancing personalized diabetes management with specific biomarker information.^70^, ^111^ This analysis is an important next step in improving quality of life for those with T1D and may help clinicians to individualize diabetes education and care.^1^

## CONFLICT OF INTEREST

Dr. Dassau reports receiving grants from JDRF, NIH, and Helmsley Charitable Trust, personal fees from Roche and Eli Lilly, patents on artificial pancreas technology, and product support from Dexcom, Insulet, Tandem, and Roche. Dr. Dassau is currently an employee and shareholder of Eli Lilly and Company. The work presented in this manuscript was performed as part of his academic appointment and is independent of his employment with Eli Lilly and Company. Dr. Doyle reports equity, licensed IP and is a member of the Scientific Advisory Board of Mode AGC. Dr. Laffel reports grant support to her institution from NIH, JDRF, Helmsley Charitable Trust, Eli Lilly and Company, Insulet, Dexcom, and Boehringer Ingelheim; she receives consulting fees unrelated to the current report from Johnson & Johnson, Sanofi, NovoNordisk, Roche, Dexcom, Insulet, Boehringer Ingelheim, ConvaTec, Medtronic, Lifescan, Laxmi, and Insulogic. Dr. Patti reports receiving grant support, provided to her institution, from NIH, Helmsely Charitable Trust, Chan Zuckerberg Foundation, and Dexcom, patents related to hypoglycemia and pump therapy for hypoglycemia, and advisory board fees from Fractyl (unrelated to the current report). Dr. Pinsker reports grant support, provided to his institution, consulting fees, and speaker fees from Tandem Diabetes Care, grant support, provided to his institution, and advisory board fees from Medtronic, grant support, provided to his institution, and consulting fees from Eli Lilly, grant support and supplies, provided to his institution, from Insulet, and supplies, provided to his institution, from Dexcom.

### PEER REVIEW

The peer review history for this article is available at https://publons.com/publon/10.1002/btm2.10201.
